# Formulation and In Vitro Characterization of a Vacuum-Dried Drug–Polymer Thin Film for Intranasal Application

**DOI:** 10.3390/polym14142954

**Published:** 2022-07-21

**Authors:** Daisuke Inoue, Ayari Yamashita, Hideto To

**Affiliations:** 1 Department of Medical Pharmaceutics, School of Pharmacy and Pharmaceutical Sciences, University of Toyama, 2630 Sugitani, Toyama 930-0194, Japan; hideto-to@pha.u-toyama.ac.jp; 2 Molecular Pharmaceutics Laboratory, College of Pharmaceutical Sciences, Ritsumeikan University, 1-1-1 Noji-higashi, Kusatsu 525-8577, Japan; fj215023@st.kobepharma-u.ac.jp; 3 Laboratory of Pharmaceutical Technology, Kobe Pharmaceutical University, 4-19-1 Motoyamakita-machi, Higashinada, Kobe 658-8558, Japan

**Keywords:** nasal formulation, film, vacuum drying, amorphous formulation, polymer, dissolution, anti-inflammatory agents

## Abstract

Intranasal drug applications show significant therapeutic potential for diverse pharmaceutical modalities. Because the formulation applied to the nasal cavity is discharged to the pharyngeal side by mucociliary clearance, the formulation should be dissolved effectively in a limited amount of mucus within its retention time in the nasal cavity. In this study, to develop novel formulations with improved dissolution behavior and compatibility with the intranasal environment, a thin-film formulation including drug and polymer was prepared using a vacuum-drying method. The poorly water-soluble drugs ketoprofen, flurbiprofen, ibuprofen, and loxoprofen were dissolved in a solvent comprising water and methanol, and evaporated to obtain a thin film. Physical analyses using differential scanning calorimetry (DSC), powder X-ray diffraction analysis (PXRD), and scanning electron microscopy SEM revealed that the formulations were amorphized in the film. The dissolution behavior of the drugs was investigated using an in vitro evaluation system that mimicked the intranasal physiological environment. The amorphization of drugs formulated with polymers into thin films using the vacuum-drying method improved the dissolution rate in artificial nasal fluid. Therefore, the thin film developed in this study can be safely and effectively used for intranasal drug application.

## 1. Introduction

The intranasal route is used as an alternative to oral administration because of its favorable membrane permeability through the nasal mucosa avoiding hepatic first-pass metabolism [1,2]. Intranasal drug administration may be used for a wide range of drug delivery systems. Various active pharmaceutical ingredients (APIs) have been recently developed, including medium-sized molecules such as peptides and nucleic acids, and macromolecules such as proteins and antibodies. These medium- and large-sized molecule APIs have found increasing use as novel biomolecular entities in particular [3]. These molecules can enter systemic circulation non-invasively via intranasal application, as they are more easily absorbed through the nasal mucosa [4]. The bioavailabilities of peptides and proteins following nasal application can reach several percentage points without any permeation enhancers. For example, the bioavailabilities of growth hormone releasing hormone (GHRH 1-29, 3358 Da) is 3–5% [5], salmon calcitonin (3432 Da) 3% [4], parathyroid hormone (PTH_1-34_, 4118 Da) 2% [6], insulin (5808 Da) 0.3% [7], and hirudin-2 (6900 Da) 2.14% [8]. The nasal administration route may be an effective alternative for non-invasive delivery of such APIs with a molecular weight around ten times larger than the 500 Da allowed for orally active drugs stated in Lipinski’s rule of five [9]. Another advantage of intranasal administration is the direct access of drugs applied in the nasal cavity to the brain, via the peripheral regions of the olfactory and trigeminal nerves that extend from the brain to the nasal cavity [10]. Drug delivery from the nose to the brain is also an effective route to regions of the central nervous system (CNS) for many types of drugs such as peptides, proteins, gene vectors, and stem cells [11]. In particular, peptides and proteins have been actively investigated in this regard [12], and some have even been used in clinical studies focusing on CNS diseases, including insulin for Alzheimer’s disease [13], oxytocin for autism spectrum disorder [14,15], melanocortin for obesity [16], and hypocretin-I for narcolepsy [17].

Drugs applied to the nasal cavity are quickly translocated to the pharynx by nasal mucociliary clearance (MC) [18,19]. Typically, more than 90% of the drug applied in the intranasal solution is cleared to the extranasal region within 30 to 60 min [20,21]. Nasal MC is a determining factor of nasal residence time, and is thus closely related to drug absorption through the nasal mucosa [22,23]. An effective dissolution process is critical for efficient drug delivery. MC is based on the interaction between ciliary beating and nasal mucus, and is maintained under regular physiological ciliary beat frequency and mucus composition. The nasal MC becomes dysfunctional if the interaction between cilia and mucus is disrupted, and so foreign materials in the nasal cavity cannot be removed. Transient or reversible inhibition of MC function is often considered an effective strategy for development of nasal formulations for efficient drug delivery, as it prolongs nasal residence time [24,25,26,27]. The balance between the reversible inhibition of MC function and the improvement in the dissolution of APIs from the solid formulation is important for efficient drug absorption from nasally administered solid formulations. In the physiological intranasal environment, the solid formulation is dissolved into the nasal mucus, releasing the APIs, which permeate through the mucosal membrane. Therefore, it is a challenge to provide a quantitative evaluation of the dissolution behavior of APIs in nasal mucus under physiological conditions. In order to design a novel nasal solid drug formulation, in this study we focused on the dissolution process and evaluated the dissolution behavior of APIs from the solid formulation in the nasal cavity in vitro by mimicking a nasal physiological environment.

The improvement of solubility in nasal mucus is essential for APIs with poor water solubility, since only a small amount of water is present in the nasal cavity. Here, we prepared solid formulations of several anti-inflammatory model drugs, including ketoprofen, flurbiprofen, ibuprofen, and loxoprofen, using a polymer to improve drug solubility in nasal mucosa. From the evidence of the safety tests in vivo, hydroxypropyl methyl cellulose (HPMC), polyvinylpyrrolidone (PVP), and alginic acid (Alg), widely used for solid dosage forms, were used as the additional polymer. Alg was selected because it is a highly biocompatible polymer. Thin films of APIs with a polymer were formulated using a vacuum-drying method. The dissolution behaviors of APIs from the film formulations were investigated using an in vitro evaluation system that mimicked the intranasal physiological environment. The potential of thin-film formulations as dosage forms for nasal application was also assessed.

## 2. Materials and Methods

### 2.1. Materials

Methanol, polyvinylpyrrolidone (K30; PVP), ketoprofen (KTP), flurbiprofen (FBP), ibuprofen (IBP), loxoprofen (LXP), mucin (from porcine stomach), phosphate-buffered saline (PBS), and trifluoroacetic acid were purchased from FUJIFILM Wako Pure Chemical Corporation (Osaka, Japan). Alginic acid sodium salt (Alg, from brown algae with low viscosity) was purchased from Sigma-Aldrich (St. Louis, MO, USA). Hydroxypropyl methyl cellulose HPMC (TC-5^®^ M) was purchased from Shin-Etsu Chemical Co. Ltd. (Tokyo, Japan). All other chemicals used were of reagent grade.

### 2.2. Vacuum Drying

Vacuum foam drying was used to prepare thin film formulations of poorly water-soluble drugs, as this method requires no heating and achieves a high degree of drying generally preferred when combustible organic solvents are used [28]. Anti-inflammatory agents KTP, FBP, IBP, and LXP were selected as poorly water-soluble active pharmaceutical ingredients (APIs). The API solutions including the polymer were prepared by dissolving the API and the respective polymer in a mixture of water and methanol at suitable mixing ratios for each API. The optimal mixing ratio was determined from the solubility of the drug and the polymer in the solvent, which was obtained by gradually changing the ratio of water and methanol (Figure S1 in Supplementary Materials). Accordingly, pure methanol was used as solvent for PVP. A mixture of water and methanol at a ratio of 1:9 was used as solvent for HPMC. For solutions including Alg, mixed solvents were used at mixing ratios based on the solubility characteristics of each API. The final water:methanol ratios were determined to be 60:40 for KTP, 50:50 for FBP, 60:40 for IBP, and 70:30 for LXP. APIs and polymer were dissolved in methanol and water, respectively. Then, both solutions were mixed at appropriate ratios for each composition. APIs and polymer were added to a final concentration of 1 *w*/*v*%, except for the control formulation which did not include the polymer (2 *w*/*v*% as API). The composition of each formulation is summarized in Table 1. The solvents of the test solutions of API with polymer (1 mL) were evaporated using a centrifugal evaporator (CC-105 with a TU-500 system, Tommy Digital Biology, Tokyo, Japan) under a constant rotation speed of 1700 rpm at room temperature (22 to 25 °C). The test solution was finally defoamed using a needle (27G, Terumo Corporation, Tokyo, Japan) where foaming was observed during the drying process. The sample was then dried until the solvent was completely removed. In line with previous studies [28], the remaining solvent in the tube was weighed on an analytical balance during the drying process, and the time at which the amount of remaining solvent was 1% or less, or no more decrease in solvent was observed, was defined as the end of drying. The drying time differed for each combination of drug and solvent used. The drying process was observed every 2 h by stopping the centrifugation. If foaming was observed, a puncture was performed; if solvent remained, the centrifuge-induced evaporation was continued until completely dry films were obtained.

### 2.3. Physical Analysis of Film Formulations 

#### 2.3.1. Differential Scanning Calorimetry (DSC)

Thermal analysis was conducted by performing differential scanning calorimetry (DSC) measurements on APIs and film formulations (DSC 60 plus, Shimadzu Corporation, Kyoto, Japan). Briefly, the samples were placed on an aluminum pan, sealed, and pinholes were made. Under nitrogen atmosphere with an air flow rate of 50 mL/min, samples were heated at a scanning rate of 10 °C/min from 40 to 200 °C, except for samples including Alg, which were heated from 40 to 300 °C in a single measurement cycle.

#### 2.3.2. Powder X-ray Diffraction Analysis (PXRD)

Powder X-ray diffraction (PXRD) analysis of the APIs and film formulations was performed using a diffractometer (Ultima IV, Rigaku Corporation, Tokyo, Japan) using Cu Kα radiation (40 mA, 40 kV). A nondiffraction plate was used as the sample holder. Data were collected from 5 to 35° (2*θ*) at a scanning speed of 10°/min (step size: 0.02°).

#### 2.3.3. Scanning Electron Microscopy (SEM)

The structures of the surfaces of APIs and film formulations were visualized using a scanning electron microscope (SEM) (TM-1000, Hitachi, Tokyo, Japan). The samples were adhered onto carbon double-stick tape, and imaged after Cu sputtering for 30 s.

### 2.4. Preparation of Artificial Nasal Fluid (ANF) 

Artificial nasal fluid (ANF) is used as an alternative to nasal mucus. ANF was prepared according to a previously reported method [29] with minor modifications. Briefly, mucin was suspended in PBS at a concentration of 4 *w*/*v*%, and the suspension was stirred for 24 h. The suspension was then centrifuged at 16,100× *g* for 20 min twice to remove insoluble debris. The obtained supernatant was stored at 4 °C, and used as ANF. Before using ANF for the experiment, the pH was adjusted to 6.4 by adding NaOH, which is comparable to physiological nasal mucus [30]. 

### 2.5. In Vitro Evaluation for Drug Dissolution in Nasal Mucus 

#### 2.5.1. In Vitro Evaluation System for Intranasal Dissolution of Solid Formulation

**Figure 1:**
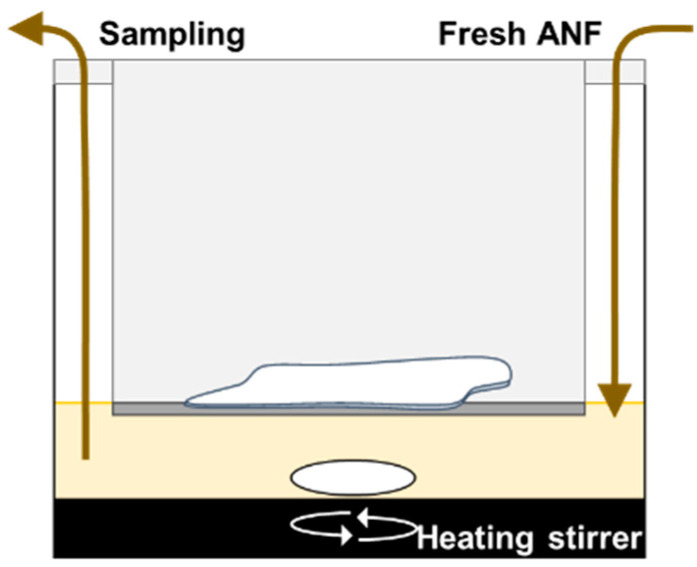
Schematic representation of an in vitro evaluation system for drug dissolution in nasal mucus in the intranasal environment. Artificial nasal fluid (ANF, derived from 4% mucin in PBS; pH6.4; 0.7 mL) was used to mimic nasal mucus, and maintained at 30 °C using a heating stirrer. Film (20 mm square) was applied onto the surface of ANF. ANF samples (10 μL) were taken, and fresh ANF was added after sampling at the appropriate intervals.

We developed an in vitro evaluation system for drug dissolution in nasal mucus that can estimate the solid dissolution process under the physiological conditions of the intranasal environment. A schematic representation of the in vitro system is shown in Figure 1. Based on previous findings on the morphological parameters of the nasal cavity (Table 2) [30,31,32,33], the experimental conditions were determined such that the volume of nasal mucus relative to the formulation dose applied in vivo was of the same order as that observed under physiological conditions. Specifically, the film was cut into 20 mm square portions, and applied onto the surface of the ANF. The ANF was heated, and maintained at 30 °C (the physiological temperature of nasal mucus) using a heating stirrer (vial hot stirrer, HSH-10VA, AS ONE Corporation, Osaka, Japan). To evaluate the drug dissolution behavior of the ANF, a cell culture insert (12 wells with a pore size of 3.0 μm, Falcon^®^, Corning Incorporated, NJ, USA) was placed in the ANF. The filter of the insert was in contact with the ANF surface. A square piece of film (with 20 mm side length) was applied onto the insert, and the ANF was continuously stirred with a magnetic stirrer bar. After applying the film, 10 μL of ANF was sampled at appropriate time points (1, 3, 5, 10, 20, 30, 45, 60, 90, and 120 min), and the same volume of ANF was added after each sampling. The samples were kept frozen until further analysis. 

#### 2.5.2. Sample Treatment

ANF samples (10 μL) were mixed with 90 μL of methanol, vortexed for 15 s, and centrifuged at 16,100× *g* for 10 min for deproteinization. The resulting supernatant was then used for quantification. The API concentrations in the samples were determined using HPLC (Agilent 1260/1290, Agilent technologies, CA, USA) analysis. LC separation was achieved using a C18 column (CAPCELL PAK C18 MG-II type, 3 μm, 4.6 mm i.d., 35 mm length, Osaka Soda, Osaka, Japan) at 40 °C with an isocratic flow of mobile phase (0.1% trifluoroacetic acid in water/0.1% trifluoroacetic acid in acetonitrile). The flow rate of the mobile phase was maintained at 1 mL/min. The analytical conditions, limit of detection (LOD), and limit of quantification (LOQ) for HPLC on the APIs are listed in Table 3.

#### 2.5.3. Dissolution Rate

The dissolution rates of the APIs from the film formulations were obtained from their dissolution behavior over time. To evaluate dissolution in detail, two values were calculated, the dissolution rate in the initial phase (*k*_dis(int)_) and the average dissolution rate until 120 min (*k*_dis(mean)_). The value obtained by calculating the varied rate from the slope between the two sampling points in the dissolution profile was used as the dissolution rate between the two time points. The initial dissolution rate (*k*_dis(int)_) was calculated as the average value from 1 to 5 min, and the average dissolution rate (*k*_dis(mean)_) was calculated from 5 to 120 min.

### 2.6. Statistical Analysis 

Results are expressed as the mean ± SEM. Statistical significance was determined using IBM SPSS software (IBM SPSS Statistics 27, IBM Corporation, New York, NY, USA) based on one-way analysis of variance. 

## 3. Results

### 3.1. Characterization of Vacuum Dried Thin Films

Uniform transparent pale films of consistent thickness were obtained by vacuum drying with centrifugal evaporation. DSC, PXRD, and SEM analyses were performed to assess the physical properties of the film formulations.

#### 3.1.1. DSC

**Figure 2:**
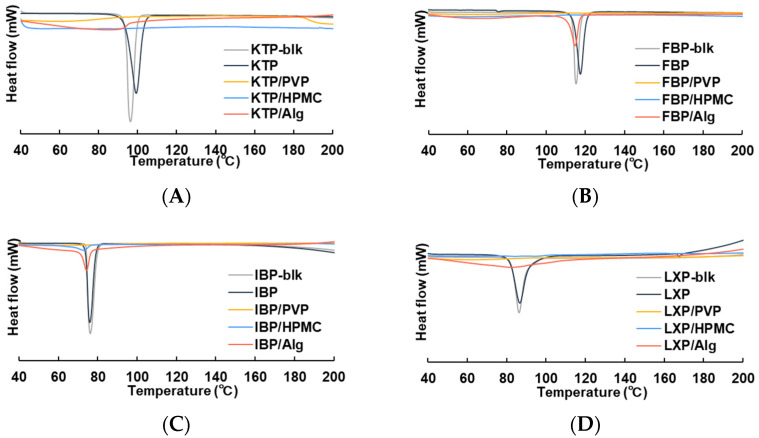
DSC thermograms of film formulations for (**A**) KTP, (**B**) FBP, (**C**) IBP, and (**D**) LXP. Thermograms are shown for API bulk (gray), control solid formulation derived from API alone (black), film formulation of API with PVP (yellow), film formulation of API with HPMC (blue), and film formulation of API with Alg (orange).

The crystal characteristics of the APIs in the thin film after vacuum drying were analyzed using DSC (Figure 2). In the control formulations prepared with API alone, endothermic peaks were observed at 99.2 °C for KTP, 117.5 °C for FBP, 76.0 °C for IBP, and at 86.7 °C for LXP. These temperature levels correspond to the melting point of each API crystalline. For all the tested drugs, APIs with a polymer were amorphized in the thin film by vacuum drying. Upon addition of PVP, the endothermic peak derived from the API crystals disappeared completely. In the HPMC formulations, the peak corresponding to API crystalline could not be obtained for KTP, FBP, or LXP, whereas a slight peak was observed at 72.9 °C for IBP. Meanwhile, the API crystalline peaks for Alg formulations were observed at 114.7 °C and 74.3 °C for FBP and IBP, respectively. No clear peak was obtained for KTP or LXP.

#### 3.1.2. PXRD

**Figure 3:**
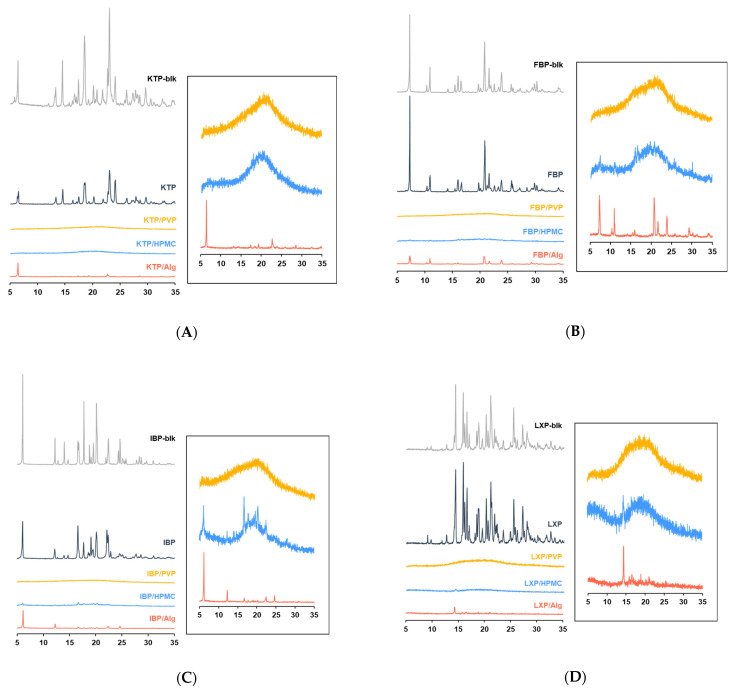
Powder X-ray diffraction (PXRD) spectra of film formulations for (**A**) KTP, (**B**) FBP, (**C**) IBP, and (**D**) LXP. Spectra indicated for API bulk (gray), control solid formulation derived from API alone (black), film formulation of API with PVP (yellow), film formulation of API with HPMC (blue), and film formulation of API with Alg (orange). The right panels show the enlarged spectra.

To clarify the precise crystalline morphology of the thin film formulation, PXRD was performed. The PXRD spectra are shown in Figure 3. In all APIs, the control formulations were found to retain the crystal phase of the bulk, since all peaks of the film coincided with the peaks observed in the bulk. Halo patterns were observed in the PVP formulations for all APIs, indicating that the film was completely amorphous. For HPMC formulations, a slight peak was obtained for some spectra in the IBP film, while the other films were shown to be amorphized completely. Several spectra were observed for all APIs with Alg formulations, suggesting that the films containing Alg were partially amorphized and retained the crystal forms of APIs. 

#### 3.1.3. SEM

**Figure 4:**
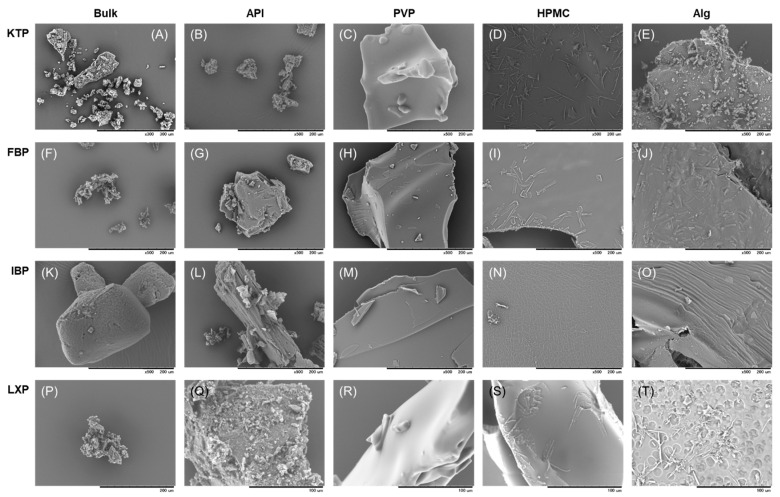
SEM images of API bulk and film formulations for (**A**–**E**) KTP, (**F**–**J**) FBP, (**K**–**O**) IBP, and (**P**–**T**) LXP. Images are shown for (**A**,**F**,**K**,**P**) API bulk, (**B**,**G**,**L**,**Q**) control solid formulation derived from API alone, (**C**,**H**,**M**,**R**) film formulation of API with PVP, (**D**,**I**,**N**,**S**) film formulation of API with HPMC, and (**E**,**J**,**O**,**T**) film formulation of API with Alg.

The surfaces of the vacuum-dried film formulations were inspected using SEM. SEM images are shown in Figure 4. KTP, FBP, and LXP were fine powders, whereas IBP was a bulk powder with a relatively large diameter. The control formulations prepared with API alone did not form a film: fine crystals were obtained by vacuum drying. In PVP formulations, a film with a smooth surface was obtained for all APIs. In the HPMC formulations, a needle-shaped crystalline structure was found to slightly adhere onto the film surface with KTP, FBP, and LXP, whereas a reticulated crystal structure was formed for IBP. In contrast, a sheet with a rough surface including streaks, chips, pores, and needle-like crystal structures, was obtained for Alg formulations.

### 3.2. Dissolution of APIs from Vacuum Dried Film in ANF

**Figure 5:**
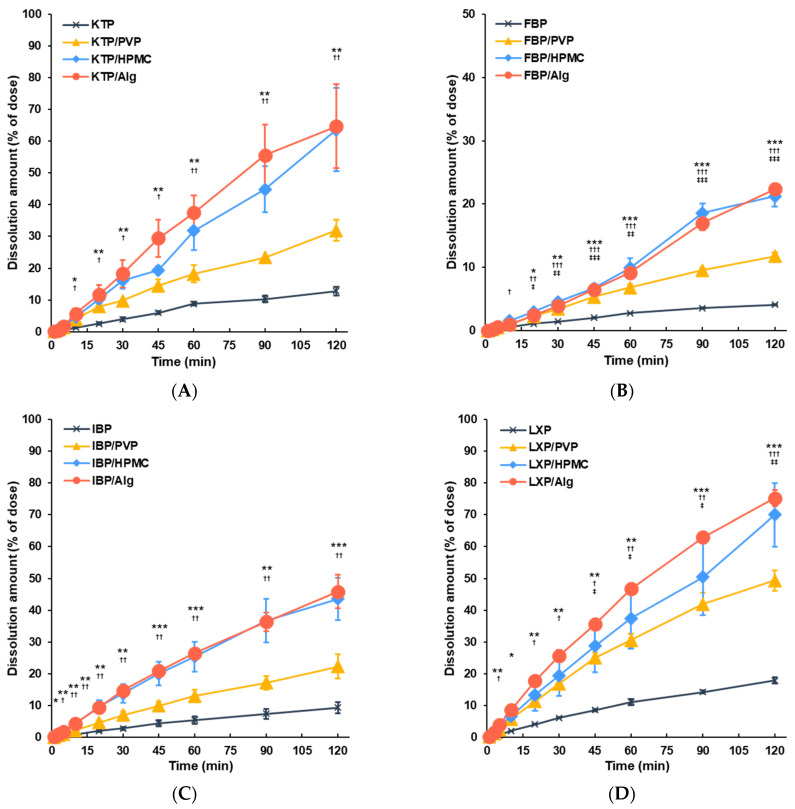
Dissolution profiles of film formulations for (**A**) KTP, (**B**) FBP, (**C**) IBP, and (**D**) LXP. Time profiles are shown for the control solid formulation derived from API alone (black), film formulation of API with PVP (yellow), film formulation of API with HPMC (blue), and film formulation of API with Alg (orange). Data are expressed as the mean ± SEM (*n* = 3–5). Statistical significances are represented as triple, double, and single symbols for *p* < 0.001, *p* < 0.01, and *p* < 0.05, respectively, compared with each condition; *, API/Alg vs. API; †, API/HPMC vs. API; ‡, API/PVP vs. API.

In vitro evaluation of the drug dissolution behavior in ANF was performed to obtain the dissolution profiles of APIs in ANF, a fluid that artificially reproduces the nasal mucus environment. The time profiles for the quantities of APIs dissolved in ANF are shown in Figure 5. The APIs of control formulations prepared with API alone were dissolved slowly in ANF in over 120 min: 12.8% for KTP, 4.1% for FBP, 9.4% for IBP, and 17.9% for LXP, owing to the poor water solubility of these APIs. The dissolution rates of the APIs for all film formulations were improved by the addition of polymers. The dissolved amounts of APIs in PVP formulations over 120 min were significantly increased to 31.9% for KTP, 11.8% for FBP, 49.3% for LXP, and 22.2% for IBP (which was not statistically significant). The effects of improving the dissolution rates of APIs were observed in HPMC and Alg formulations, with comparable effects for both polymers: rates in films with HPMC and Alg applied for 120 min were significantly increased to 63.6 and 64.7% for KTP, 21.1 and 22.4% for FBP, 43.6 and 45.9% for IBP, and 70.0 and 75.2% for LXP, respectively.

**Figure 6:**
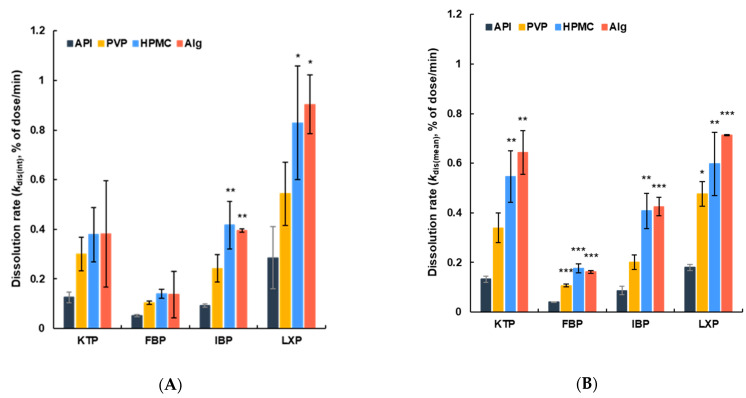
Dissolution rate constant of film formulations. (**A**) The initial dissolution rate from 1 to 5 min (*k*_dis(int)_) and (**B**) the mean dissolution rate from 5 to 120 min (*k*_dis(mean)_). Columns show data for the control solid formulation derived from API alone (black), film formulation of API with PVP (yellow), film formulation of API with HPMC (blue), and film formulation of API with Alg (orange). Data are expressed as the mean ± SEM (*n* = 3–5). Statistical significance is denoted with ***, *p* < 0.001; **, *p* < 0.01; *, *p* < 0.05 compared to respective control formulations.

The dissolved amount per unit time (i.e., the average rate of dissolution) was calculated from the increase between two adjacent sampling points (*k*_dis_). The dissolution rate (*k*_dis_) was found to increase for 10–15 min after film application, and remained constant until approximately 60 min, and then gradually decreased from 60 to 120 min (Figure S2 in Supplementary Materials). Therefore, two different average values of *k*_dis_ were calculated, the initial rate (*k*_dis(int)_) from 1 to 5 min and the mean rate (*k*_dis(mean)_) from 5 to 120 min, to evaluate the dissolution behavior (Figure 6). *k*_dis(int)_ values were found to increase from 2 to 2.5-fold in PVP-added film formulations compared with the control film prepared with API alone. Initial rates were determined of 2.4 for KTP, 2.0 for FBP, 2.6 for IBP, and 1.9 for LXP. This increase was even higher with film formulations prepared with HPMC and Alg, with *k*_dis(int)_ being approximately 3 to 4-fold higher than that of the control formulations. Accordingly, the ratios of dissolution rates of KTP, FBP, IBP, and LXP were 3.0, 2.7, 4.5, and 2.9 for HPMC, and 3.0, 2.7, 4.5, and 3.2 for Alg-containing film formulations, respectively. Similar effects on dissolution rate were obtained depending on the polymer species used to prepare the film formulation, and regardless of the API used. The *k*_dis(mean)_ showed a similar trend to that of the initial rate (*k*_dis(int)_). Compared to control formulations, dissolution rates were found to increase by approximately 2.5 fold for PVP-containing films (2.6 for KTP, 2.7 for FBP, 2.3 for IBP, and 2.6 for LXP), whereas the rates increased from 4 to 5-fold for HPMC and Alg-containing films (4.1 and 4.8 for KTP, 4.4 and 4.1 for FBP, 4.7 and 4.9 for IBP, 3.3 and 4.4 for LXP, for HPMC and Alg, respectively). Nevertheless, these increases for films of KTP and IBP containing PVP were not statistically significant.

## 4. Discussion

We prepared thin-film formulations of poorly water-soluble APIs using the vacuum-drying method, and assessed the applicability of the film for nasal formulations. Film formulations for nasal application have not yet been used in clinical contexts, and only few studies have been conducted on thin films. A mucoadhesive film for topical application into the nasal cavity was recently prepared by Laffleur [34], and a fast-dissolving film of insulin for intranasal application was developed by Mohamad et al. [35]. These studies highlight the potential of film dosage forms for nasal formulations. In our study, thin-film formulations of poorly water-soluble APIs mixed with polymers were developed to further explore the potential of thin films to increase the effectiveness of drug administration. The dissolution rates of all studied APIs (model drugs) increased significantly in the film formulations (Figure 6), indicating that the thin film prepared by vacuum drying may be a useful dosage form for intranasal formulations. From the perspective of the clinical applications of thin-film formulations, because the film formulations prepared were very thin, they can be attached directly to the nasal cavity. Therefore, it is unlikely that the application of the film would increase patient distress. Regarding the solution or solid dosage forms, including powders, because the drug applied intranasally is cleared to the extranasal region by nasal mucociliary clearance, the intranasal residence time of the drug should be considered for nasal formulations. In contrast, because the thin film is in contact with the nasal mucosa over a relatively large surface area, it is considered that the effect of mucociliary clearance can be suppressed. 

The drug and the polymer were both dissolved or suspended in a mixed solvent of water and methanol, within the range of concentrations used in this study (Table 1). Since foaming of the solution was observed during the vacuum drying process, a puncture was made using a needle to remove the foam from the solution by allowing the solvent to completely evaporate, as implemented previously by Takeda et al. [28]. A transparent or pale white dry thin film was obtained by vacuum drying via centrifugal evaporation. Various formulation techniques such as spray drying [24,36,37,38] and freeze drying [39,40,41,42,43] can be applied for formulation of nasal solid dosage forms. A vacuum-drying method was used to prepare a solid dosage form in this study. Vacuum drying is considered especially suitable for solidifying a mixture of drug and polymer solution, as it enables evaporation of organic solvents without heating [28]. In addition, drying without heating may be useful for preparation of solid formulations of biological drugs such as peptides. Vacuum drying thus also has the potential to be widely used for nasal formulations. Here, we successfully prepared thin film formulations in which a poorly water-soluble drug and a polymer were mixed without any surfactants using a simple vacuum-drying procedure. However, further investigations are needed to assess its applicability to heat-sensitive biological compounds such as peptides and antibodies.

Each API and polymer were completely dissolved in methanol and water. Then, the two solutions were mixed to prepare a “mixed solution” for vacuum drying. The optimal mixing ratio of the API and polymer in the mixed solution was determined (Figure S1). Since APIs with poor water solubility were selected as model drugs, the solubility of the API decreased as the water ratio increased, and the API precipitated in the mixed solution. Therefore, the composition of the mixed solution was adjusted to achieve the maximum methanol amount. For the solution with PVP, pure methanol (water:methanol ratio of 0:100) was used for all APIs, as both API and PVP were completely dissolved in methanol. Likewise, a mixed solution (10:90) was used to dissolve HPMC, since all APIs and the polymer were completely dissolved in only this mixed solution. Meanwhile, the API and the polymer could not be completely dissolved in any of the mixed Alg-containing solutions. Thus, mixed suspensions that included uniformly dispersed particles according to the APIs’ and polymers’ solubility were prepared at different mixing ratios (water:methanol 60:40 for KTP, 50:50 for FBP, 60:40 for IBP, and 70:30 for LXP, respectively; Table 1). The thickness of films is an important factor affecting their dissolution behavior. However, this could not be measured because the films were too thin. The HPMC formulation was thinner than the Alg formulation, while the PVP formulation appeared to be the thickest film. Because the contact area of the film and ANF was set to 20 mm^2^ to achieve similar contact areas, the formulation dose in the film was 2 mg for the HPMC and Alg formulations and 10 mg for the PVP formulation. Owing to differences in the applied dose in the films, the dissolution rate of PVP may have decreased compared with the other films. Therefore, the effect of improving the dissolution behavior by adding a polymer was confirmed in this study, but the polymer type used could not be optimized. In future studies, it will be necessary to study a method to prepare a uniform film formulation and to optimize the components, including the polymers used, to prepare films that exhibit high dissolution behavior.

The API was stabilized by adding the polymer to the solution after dissolution [44,45,46]. Thus, the product obtained following vacuum drying could exist as an amorphous solid dispersion (ASD). The endothermic peaks of APIs disappeared in DSC when HPMC or PVP was used as additive (Figure 2). A “halo” pattern, typical in the amorphous state, was observed in PXRD (Figure 3). In addition, although a weak endothermic peak associated with the API crystal was observed in DSC for FBP and IBP, most of the API was found to be amorphized due to the presence of Alg (Figure 2). For PXRD, similar results were obtained, and slight crystalline peaks appeared in films with Alg (Figure 3). In contrast, an endothermic peak of API crystalline was observed in all control formulations prepared with API alone (Figure 3), suggesting that a dried solid obtained in the control formulations was formed while maintaining the crystal morphology of the API bulk. These results suggest that centrifugal vacuum drying is a useful method for preparing solid ASD formulations from a mixture of API and polymers. Thermal processing technologies are often used for manufacturing of ASD for solid formulations [47]. Commonly utilized thermal processing methods for bulk and powder manufacturing include hot-melt extrusion (HME) [48,49,50,51,52] and spray drying (SD) [53,54,55,56,57]. In contrast, vacuum drying does not require a heating process [28], and offers many advantages such as applicability to APIs with a high melting point or low resistance against thermal and shear stress, and the potential for processing highly viscous solutions and those of high molecular weight, which are difficult to process using HME and SD [58,59]. Vacuum drying may thus be an optimal drying method for nasal formulations, which are suitable for various drug modalities such as peptides, proteins, nucleic acids, and antibodies. Large-molecular-weight drugs and thermally unstable compounds were not included in this study. The applicability of the vacuum drying method for nasal formulations of medium- or large-molecular-weight drugs will be addressed in future studies. 

The surfaces of the vacuum-dried film formulations were inspected using SEM (Figure 4). For all APIs, PVP films with smooth surfaces were obtained (Figure 4C,H,M,R). Since no crystalline peaks were observed in PXRD, it was apparent that amorphized films with a uniform shape were obtained by mixing APIs and PVP. In the HPMC formulations, a needle-shaped crystalline structure was found on the film surface for KTP, FBP, and LXP, whereas a reticulated form was obtained with IBP (Figure 4D,I,N,S). It is possible that slightly residual crystalline influenced the shape of the film surface (Figure 3). In contrast, films with rough surfaces including streaks, chips, pores, and needle-like structures were obtained for Alg formulations (Figure 4E,J,O,T). Some crystalline peaks were observed for the Alg films in the PXRD analysis (Figure 3), speculating that the surface structure was roughened by the residual crystalline. However, it was shown that these fine surface structures did not significantly affect the dissolution behavior of APIs in the film, and the dissolution rate changed depending on the polymer species added (Figure 5). Although differences in surface structure were observed by SEM, it is considered that the APIs in the film were almost amorphized.

In the DSC analysis, although DSC thermograms were obtained in the temperature range of 40 °C to 200 °C, the glass transition of the amorphous materials could not be observed. Since the APIs used in this study have low glass transition temperatures (Tg) (−14 °C for KTP [60], −4.65 °C for FBP [61], −42.3 °C for IBP [62], and −45 °C for LXP [63]), it is possible that amorphous-derived Tgs were not obtained above 40 °C. Therefore, to confirm the existence of the amorphous phase, we also examined the PXRD results and used these to reach a comprehensive conclusion. The PXRD results indicated that most of the API was in the amorphous state, or a mixture of the amorphous and crystalline forms. Understanding the crystalline state of the film at the molecular level is an important factor in precisely controlling dissolution behavior, and a study investigating glass transition should be considered to optimize film formulations. Regarding storage stability, the same physical properties and dissolution behavior were observed after 14 days of storage at 25 °C; hence, we considered them to be stable for at least 14 days. It is therefore possible that the obtained films have constant storage stability that makes them usable for extended periods.

Polymers can play an important role in the dissolution of poorly water-soluble APIs. The addition of polymers can improve the apparent solubility and drug absorption of poorly water-soluble APIs by inhibiting and retarding the precipitation of supersaturated APIs [46,64]. Therefore, poorly water-soluble APIs are often stabilized at their supersaturated concentrations by addition of a polymer, leading to improved bioavailability [65]. The HPMC, PVP [60], and Alg [66] used in our study are additives that can improve the dissolution properties of poorly water-soluble APIs. Our findings show that the API was supersaturated in the solvent upon mixing with the polymer as an excipient. Thus, the solubility of APIs in the film formulations was increased, as was their amorphization during the vacuum drying process. The stabilization of the supersaturated APIs due to the presence of polymers improved the solubility of the APIs, and significantly improved the dissolution rate constant of all APIs within ANF (Figure 6). In summary, film formulations prepared by adding a polymer to the poorly water-soluble APIs improved their solubility in ANF, and a useful film formulation for nasal application with enhanced dissolution in nasal mucus was thus developed using vacuum-drying technology.

Safety and efficacy must be assessed by in vitro evaluation as the next step in the development and clinical use of film formulations for intranasal application. We plan to evaluate the biocompatibility of the formulations investigated in this study, using cultured cell lines to estimate their toxicity in the epithelial membrane and their drug permeability through the nasal mucosa. 

## 5. Conclusions

In this study, we successfully demonstrated the enhancement of drug dissolution in nasal mucus using a film formulation of poorly water-soluble APIs with polymers prepared by vacuum drying with centrifugal evaporation. The dissolution behavior of the drugs in the films was investigated using an in vitro evaluation system that mimics the intranasal physiological environment. The amorphization of drugs formulated with polymers into thin films using the vacuum-drying method improved the dissolution rate in artificial nasal fluid. Vacuum-drying technology may be useful for the preparation of solid dosage forms for nasal formulations. Since film formulations are expected to reside in the nasal cavity for a long time, films with improved solubility in nasal mucus may be the key to enabling novel applications for nasal formulations. Nevertheless, further studies are required to develop better film formulations for clinical use, including the preparation of film formulations of various pharmaceutical modalities, the development of films capable of sustained drug release, the control of drug dissolution behavior, and verification of efficacy and safety following nasal application in vivo. In forthcoming projects, in vivo studies on intranasal residence time, film dissolution, drug release in the nasal cavity, drug absorption and pharmacokinetics, and in vivo safety and tolerability will be considered.

## Figures and Tables

**Table 1:** Compositions of vacuum dried formulations.

APIs		API Concentration (*w*/*v*%)	Polymer Concentration (*w*/*v*%)			Solvent Concentration (*v*/*v*%)	
			HPMC	PVP	Alg	Water	Methanol
Ketoprofen (KTP)	Control	2	-	-	-	-	100
	HPMC	1	1	-	-	10	90
	PVP	1	-	1	-	-	100
	Alg	1	-	-	1	60	40
Flurbiprofen (FBP)	Control	2	-	-	-	-	100
	HPMC	1	1	-	-	10	90
	PVP	1	-	1	-	-	100
	Alg	1	-	-	1	50	50
Ibuprofen (IBP)	Control	2	-	-	-	-	100
	HPMC	1	1	-	-	10	90
	PVP	1	-	1	-	-	100
	Alg	1	-	-	1	60	40
Loxoprofen (LXP)	Control	2	-	-	-	-	100
	HPMC	1	1	-	-	10	90
	PVP	1	-	1	-	-	100
	Alg	1	-	-	1	70	30

**Table 2:** Morphological differences in rats and humans for nasal cavity and mucus.

Species	Length (mm)	Volume (mm^3^)	Surface Area (mm^2^)				Mucus Thickness (μm)	
			Squamous Epithelium	Respiratory Epithelium	Olfactory Epithelium	Total Surface Area	Periciliary Layer	Surface Layer
Rat	91 ± 0.3 [31]	256.7 ± 4.1 [31]	44.2 ± 5.2 [31]	623.1 ± 14.0 [31]	675.2 ± 43.0 [31]	1343.5 ± 55.0 [31]	5–10 [32]	1–10 [32]
Human	100–140 [33]	20,000 [33]	-	-	200–400 [33]	16,000 [33]	5 [30]	10–15 [30]

**Table 3:** HPLC analytical conditions for each APIs.

APIs	Mobile Phase (Acetonitrile/0.1% TFA)	UV Wavelength (nm)	Injection Volume (μL)	Retention Time (min)
KTP	50/50	254	10	1.35
FBP	60/40	220	20	1.01
IBP	60/40	244	20	0.82
LXP	35/65	223	30	1.10

## Data Availability

Not applicable.

## Supplementary Materials

The following supporting information can be downloaded at: https://www.mdpi.com/article/10.3390/polym14142954/s1, Figure S1: Images taken during mixing of solvents; Figure S2: Changes in the dissolution rate constant over time.
