# Supplementary material for: Formulation and In Vitro Characterization of a Vacuum-Dried Drug–Polymer Thin Film for Intranasal Application
Source: Polymers (Basel). 2022 Jul 21;14(14):2954. doi: 10.3390/polym14142954 (PMC9320708; doi:10.3390/polym14142954)
Supplement: Supplementary file 1 [file polymers-14-02954-s001.zip › polymers-1814680-supplementary.pdf]

Article

Formulation and In Vitro Characterization of a Vacuum-Dried Drug–Polymer Thin Film for Intranasal Application

Daisuke Inoue [1,2,*], Ayari Yamashita [2,3] and Hideto To [1]

[1] Department of Medical Pharmaceutics, School of Pharmacy and Pharmaceutical Sciences, University of Toyama, 2630 Sugitani, Toyama 930-0194, Japan; dinoue@pha.u-toyama.ac.jp (D.I.); hidetoto@pha.u-toyama.ac.jp (H.T.)
[2] Molecular Pharmaceutics Lab., College of Pharmaceutical Sciences, Ritsumeikan University, 1-1-1 Noji-higashi, Kusatsu, Shiga 525-8577, Japan
[3] Laboratory of Pharmaceutical Technology, Kobe Pharmaceutical University, 4-19-1 Motoyamakita-machi, Higashinada, Kobe 658-8558, Japan; fj215023@st.kobepharma-u.ac.jp (A.Y.)
* Correspondence: dinoue@pha.u-toyama.ac.jp

Supplementary Materials

Figure S1. Images taken during mixing of solvents.

Figure S2. Changes in dissolution rate constant over time.

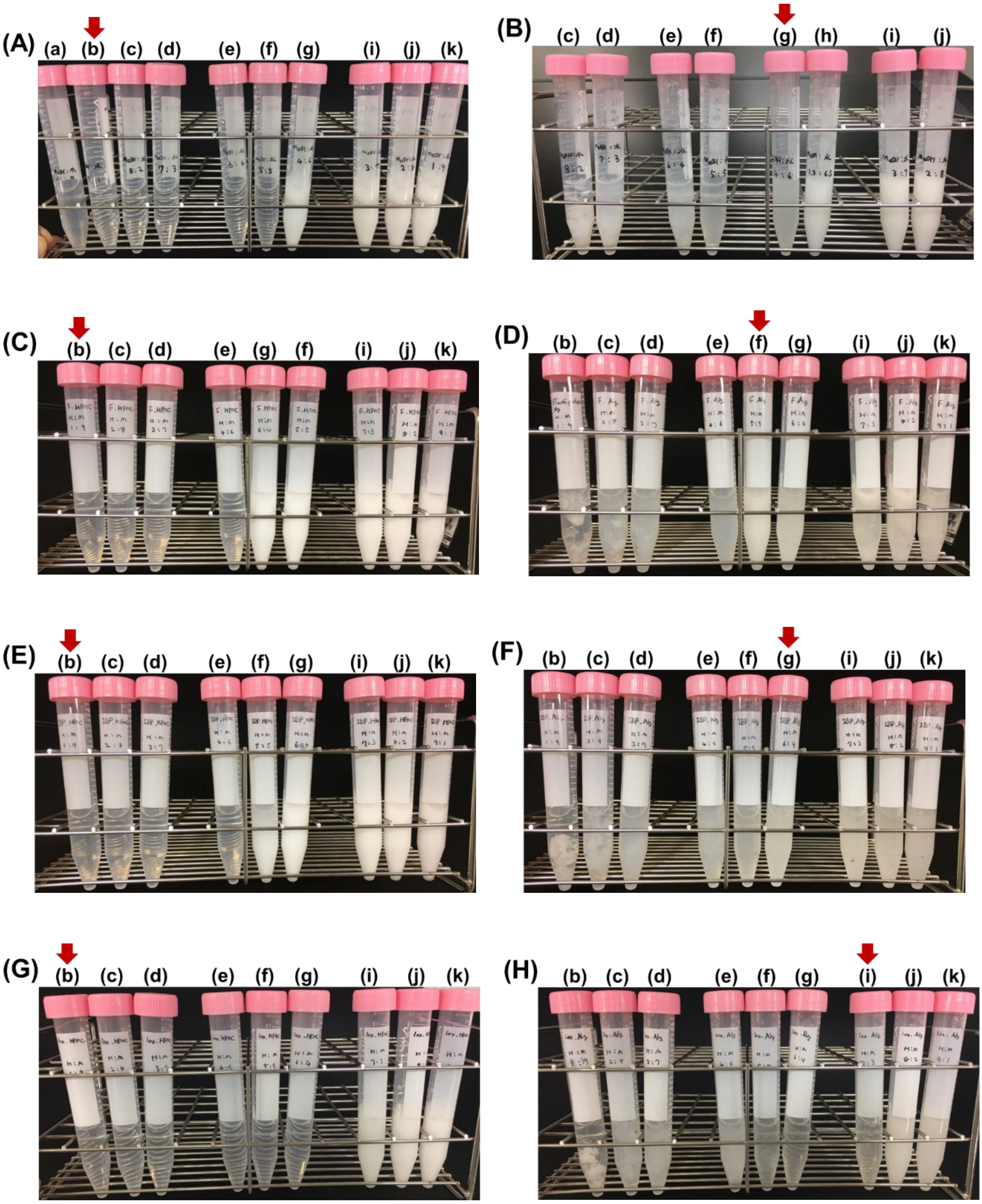

Figure S1. Images taken during mixing of solvents. API and polymer were dissolved in methanol and water, respectively. The methanol and aqueous solutions were then mixed at different methanol:water ratios:10:0 (a), 90:10 (b), 80:20 (c), 70:30 (d), 60:40 (e), 50:50 (f), 40:60 (g), 35:75 (h), 30:70 (i), 20:80 (j), and 10:90 (k) for (A) KTP/HPMC, (B) KTP/Alg, (C) FBP/HPMC, (D)FBP/Alg, (E) IBP/HPMC, (F) IBP/Alg, (G)LXP/HPMC, and (H) LXP/Alg, respectively.

Figure. S2

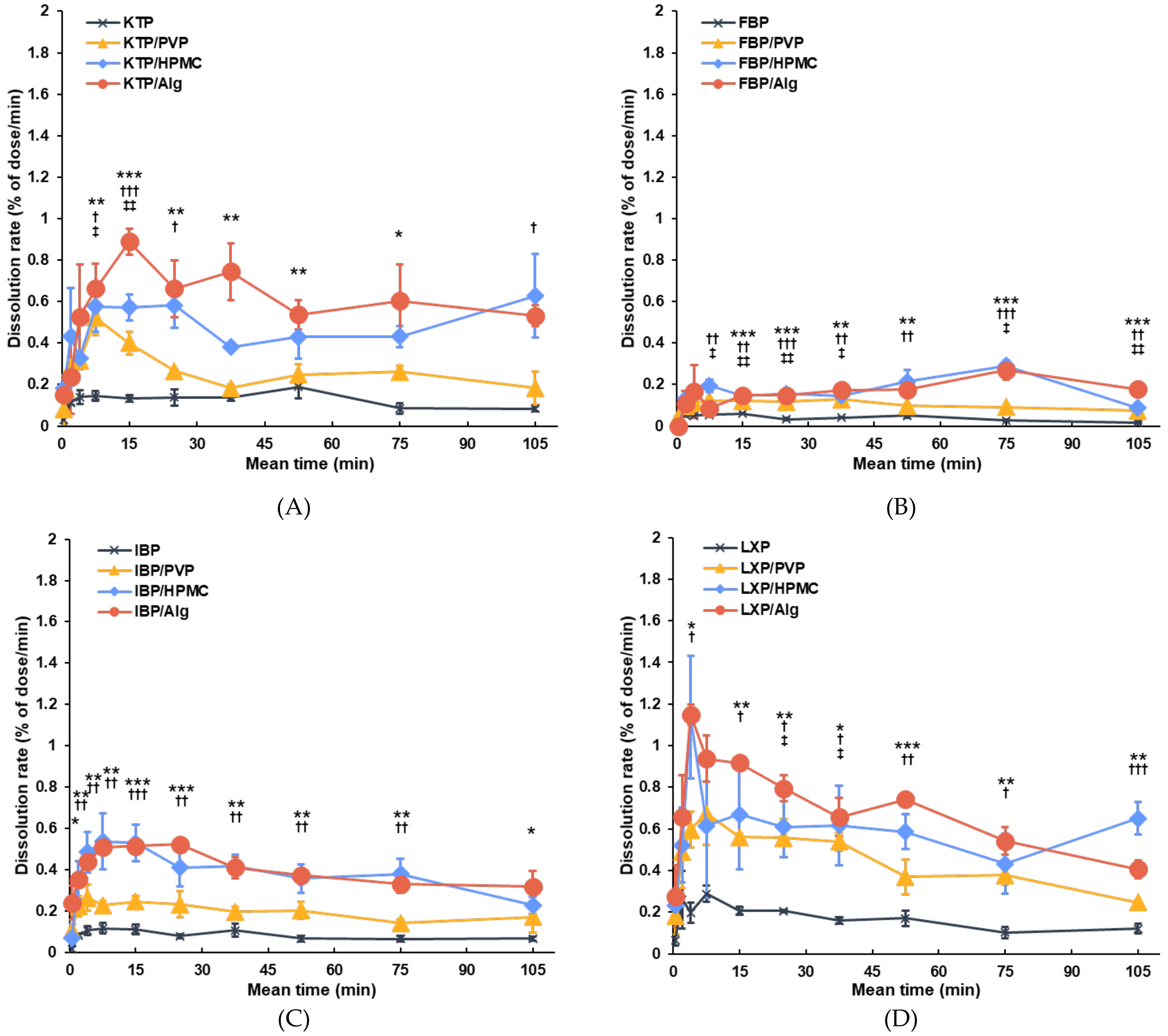

Figure S2. Changes in dissolution rate constant over time. Time profiles for (A) KTP, (B) FBP, (C) IBP, and (D) LXP are represented for the control solid formulation derived from API alone (black), film formulation of API with PVP (yellow), film formulation of API with HPMC (blue), and film formulation of API with Alg (orange). Data are expressed as the mean ± SEM (n=3–5). Statistical significance levels are represented as p<0.001, p<0.01, p<0.05, compared to each condition; *, API/Alg vs. API; †, API/HPMC vs. API; ‡, API/PVP vs. API.
